# Dental Caries in Permanent First Molar and Its Association with Carious Primary Second Molar among 6–11-Year-Old School Children in Sunsari, Nepal

**DOI:** 10.1155/2023/9192167

**Published:** 2023-03-21

**Authors:** Santosh Kumari Agrawal, Tarakant Bhagat, Ashish Shrestha

**Affiliations:** Department of Public Health Dentistry, B.P. Koirala Institute of Health Sciences, Dharan, Nepal

## Abstract

The permanent first molar (PFM) plays an essential role in maintaining the dental and overall health of an individual. It is the most susceptible tooth to dental caries due to its early eruption and location near the primary second molar in the oral cavity. We assessed the clinical status of the PFM and its association with carious primary second molars among 6–11-year-old children in Sunsari, Nepal, from January 2019 to December 2021. We recorded DMFT/DMFS and dft/dfs indices of the first permanent molar and secondary primary molar. Chi-square, logistic regression, and Spearman rank correlation (*r*_s_) were used to explore the association between carious molar lesions. Of the 655 children, only 612 had all first permanent molars. The prevalence of caries was higher in the second primary molar (70.9%) than in the PFM (38.6%). In both molars, the occlusal surface was the most commonly affected surface by dental caries. A significant association (*p* < 0.01) was found between the decayed primary second molar and the decayed PFM. A moderate but statistically significant correlation (*p* < 0.01) was found between the occurrence of dental caries in both the molars.

## 1. Introduction

Caries affecting the primary tooth is an important predictor for caries in the permanent tooth at later age [1]. An 8-year cohort study found that the risk of developing dental caries in permanent dentition increased by three times compared to noncarious primary dentition [2].

The permanent first molar (PFM) is the most caries-susceptible tooth due to its morphology and early eruption in the oral cavity [3–6]. The prevalence of caries on PFM has been reported to be 40.2%, 66.4%, and 75.5% among 6–7, 7–10, and 9–12-year-old children, respectively [5, 7, 8]. Early cavitation in PFM and its sequelae can affect children's physical growth, self-esteem, and social development due to missing or damaging teeth. The significance of PFMs cannot be overemphasized, as they form the key to occlusion. Therefore, the urgent establishment of cost-effective community preventive and promotion programs for the early detection and prevention of dental caries is important. These programs should reduce, and possibly even eliminate, the need for complex, expensive restorative treatments.

The presence of carious primary second molar increases the experience of caries of adjacent PFMs, as it is the most common deciduous tooth affected by dental caries [9]. Many studies have established the fact that carious primary teeth increase the chance of developing cavities in the PFM [9–11].

In eastern Nepal, the prevalence of dental caries among public school children was found to be 60.3% for the primary dentition and 55.6% for the permanent one [12]. However, no data are available on the caries experience of PFM in Nepalese children and its relationship with carious primary second molar. We aimed to find the prevalence of dental caries in PFMs and explore any association with carious primary second molars among children aged 6–11 years in Sunsari, Nepal.

## 2. Materials and Methods

A cross-sectional study was conducted from January 2019 to December 2021 among school children aged 6–11 years. Ethical approval for the study was obtained from the institutional review committee of B.P. Koirala Institute of Health Sciences (BPKIHS) (Ref no. 115/075/076). All together, seven schools (five public and two private) in Sunsari district were visited by the Department of Public Health Dentistry team, College of Dental Surgery, BPKIHS, Dharan, as a part of the school's oral health program. Children present during the examination were enrolled in the study, but unwilling/uncooperative children/parents/principals were excluded. A total of 655 children participated in this study. Based on the prevalence of dental caries (40.2%) in PFMs in school children in Pitesti (Romania) [7], we estimated the sample size for a 5% *α* and 10% margin of error.

(1) $$\begin{array}{c} n={\left(Z\alpha \right)}^{2}PQ/d^{2}, \end{array}$$

where *Zα* = 1.96 (when *α* is 5% for 95% confidence limits), *P* = 40.2%, *Q* = 1*−P* (59.81%), *d* = acceptable margin of error (10%).

This estimate gave a sample of 595 students; with an additional 10% nonresponse rate, the sample size was 655.

A pro forma was designed to record the personal profile of the student and DMFT/DMFS/dft/dfs scores for PFM and primary second molar to calculate the prevalence of dental caries. We used the World Health Organization [12] criteria to diagnose the presence of dental caries [12]: (a) the lesion is clinically visible and obvious, (b) the explorer tip can penetrate deep into soft yielding material, (c) the presence of discoloration or loss of translucency typical of undermined enamel, (d) when an explorer tip is placed in a pit, the fissure catches or resists removal of the tip after moderate-to-firm pressure on insertion and when there is softness at the base of the area were used to diagnose the presence of dental caries on the teeth. The oral examination was done by two trained and calibrated examiners. The dental explorer and plane mouth mirror were used to identify decayed, missing, and filled teeth under natural light with the patient sitting in or supine position in a classroom. The interexaminer reliability of two examiners was measured among 25 children of the same age group. The kappa coefficients were 0.86 for the primary and 0.77 for the permanent molar.

### 2.1. Data Handling and Statistical Analysis

All the collected data were fed into an excel sheet and analyzed using SPSS version 28.0.1.1. (SPSS, Inc., Chicago, IL, USA). Mean, standard deviation, frequency, and percentage were calculated and tabulated. Chi-square and logistic regression were used to assess the association between categorical variables. To compute association DMFT/dft score has been categorized as “no caries” (DMFT/D/dft/*d* = 0) and “presence of caries” (DMFT/D/dft/*d* ≥ 1). Spearman's correlation coefficient (*r*_s_) was computed to explore the correlation between decayed primary second and PFMs. The level of significance was taken as *p* < 0.05.

## 3. Results

The mean age (SD) of the 655 students was 9.10 (1.59) years. Males represented (*n* = 363) 59.3% of the study population. In total, 612 (93.4%) had all (four) molars in their oral cavity and were included in the analysis. Of those with unerupted PFMs, 35 children had all four unerupted, and eight children had any one of the molars unerupted.

The overall prevalence of dental caries was higher in the primary second molar (434; 70.9%) than in the PFM (236; 38.6%). The mean ± standard deviations were: DMFT 0.72 ± 1.06, DMFS 0.87 ± 1.61, dft 1.69 ± 1.44, and dfs 3.24 ± 3.91. In girls, 40.6% (*n* = 101) of PFMs were carious, whereas, in boys, caries was most predominant in primary second molars (71.9%, 261). No differences in overall caries prevalence were observed between genders (*p* > 0.05). Maxillary permanent first and primary second molars exhibited a higher number of decayed teeth than mandibular molars (Tables 1 and 2).

The distribution of dental caries was higher on the occlusal surface of both the primary second (54.1%) and the PFM (35.8%) (Table 3). Furthermore, we found that the presence of dental caries on a distal surface of the primary second molar did not significantly affect the development of dental caries on the mesial surface of the PFM (*r*_s_ = −0.02, *p* = 0.92).

We found a significant association between the presence of a decayed primary second molar and a decayed PFM (*χ*^2^ = 161.56, *p* < 0.01). Furthermore, the results showed that when the primary second molars were carious, the adjacent first permanent molars were also carious (369; 85.0%). Individuals who had carious primary molars showed a higher risk of developing caries in PFMs (OR = 11.4; 95% confidence interval 7.61–17.22; *p* < 0.01) (Table 4).

A moderate though the statistically significant correlation was found between the decayed first permanent molars and the primary second molars (*r*_s_ = 0.51, *p* < 0.01). However, both right and left second molars showed a low correlation with their neighboring PFMs (Table 5).

## 4. Discussion

The PFM is the most important tooth in the oral cavity, which plays an essential role in mastication, bearing the maximum occlusal load, and in maintaining the dental and overall health of an individual. PFMs are the first tooth to erupt in the oral cavity and are more susceptible to dental caries because of their functional and morphological characteristics. Therefore, it requires special vigilance from dental practitioners to ensure that early carious lesions are treated [13]. Our findings highlight the importance of caries prevention and treatment of PFMs and the promotion of oral health of schoolchildren.

The present study found that one-third of schoolchildren had carious PFMs, and more than half of the study population had carious primary second molars. This finding is in concordance with data reported in 2014 National Oral Health Policy [14], where 41.0% of permanent dentition was found to be carious in children aged 12–13. In Saudi Arabia [15], 24.6% of 6–9-year-old school girls had carious PFMs, which is lower than the results of the current study. Caries in schoolchildren in Romania [7] (44.0%), Jordan [16] (66.0%), and India (55.3%) [17] were more common than in Nepal.

Factors that could influence the development of carious lesions in the PFM include the presence of deep pits and fissures on the occlusal surface, the large-sized crown, which leads to the accumulation of acid produced by bacteria, and the early eruption of the tooth [5]. Early preventive programs like the application of fissure sealants, biomimetic zinc-hydroxyapatite paste, use of fluoride, provision of oral hygiene instruction, motivation and reinforcement to children regarding maintenance of oral hygiene, and regular visits to the dentist could help in reducing the prevalence of caries in PFMs. In addition to preventive measures for dental caries, there are other approaches available to treat cavitated lesions, such as conventional restorative techniques or minimally invasive techniques [18, 19].

We also showed that the upper jaw was more affected by dental caries than the lower jaw. This finding is not in alignment with the results reported from India [10] and Saudi Arabia [20], where mandibular molars were commonly affected in comparison to maxillary molars. Our higher rate of maxillary molar decay could be due to inappropriate indirect visualization leading to ineffective and difficult access to effective oral hygiene by schoolchildren [21].

Several studies reported, as we do, that dental caries in the primary second molar is a strong predictor of the appearance of caries in the PFM [9–11, 22, 23]. The positive correlation of carious primary second molars with carious PFMs was probably due to the closer location of the carious lesion and the site of food impaction with the PFM when the primary second molar is carious [23].

The occlusal surface was the most common surface affected by dental caries in both types of molars. A similar result has been reported in India [24]. A possible explanation for this finding could be the presence of anatomical structures, such as a greater number of pits and fissures that act as food retention areas.

Furthermore, the present study also shows a poor correlation between the presence of dental caries on the distal surface of the primary second molar and the mesial surface of PFM. This finding is inconsistent with a 4-year prospective study done in Greece [10]. The risk of developing dental caries on the proximal surfaces of the primary and permanent molars is low in the early period of the mixed dentition period, starting from 6 to 9 years. Afterward, the risk of dental caries increases as the child is exposed to a cariogenic environment, and the occurrence of caries is considered a cumulative process [10, 25].

The strong points of this study are the large sample size, standardized clinical examination methods, and strict quality control. There are also some constraints that could be addressed in future work: (a) inclusion of questions about children's eating and oral hygiene behaviors to help understand the reasons for the prevalence of caries in PFMs and their association with the adjacent primary second molar; (b) the cross-sectional nature of the study means that it is difficult to establish the temporal causation of dental caries seen in the study. Longitudinal studies are needed for this.

## 5. Conclusion

The prevalence of dental caries was high in both PFMs and primary second molars of schoolchildren aged 6–11 years in Nepal. A significant association was found between carious primary second molar and adjacent PFMs. Furthermore, we showed that the presence of a carious lesion on the distal surface of the primary second molar does not increase the risk of developing lesions on the mesial surface in PFMs.

## Figures and Tables

**Table 1:** Number (%) of children with and without caries on permanent first and primary second molars (*n* = 612).

Tooth	Jaw	Decayed	Sound
Primary second molar	Maxilla	341 (55.7)	271 (44.3)
	Mandible	336 (54.9)	276 (45.1)

Permanent first molar	Maxilla	341 (55.7)	271 (44.3)
	Mandible	198 (32.3)	414 (67.6)

**Table 2:** Gender-wise caries experience among participating children (*n* = 612).

	Male *n* (%)	Female *n* (%)	*p* value^a^
Primary second molar	261 (71.9)	173 (69.5)	0.517
Permanent first molar	135 (37.2)	101 (40.6)	0.40

^a^ *χ* ^2^ test.

**Table 3:** Surfaces-wise distribution of dental caries on molars (*n* = 612).

Surfaces	Primary second molar *n* (%)	Permanent first molar *n* (%)
Occlusal	331 (54.1)	219 (35.8)
Buccal	88 (14.4)	38 (6.2)
Lingual	123 (20.1)	24 (3.9)
Mesial	221 (36.1)	21 (3.4)
Distal	148 (24.2)	19 (3.1)

**Table 4:** Association of decayed primary second and permanent first molar.

Primary second molar	Permanent first molar		*p* value^c^	Crude OR^d^	(95% confidence interval)
	*D* ^a^ = 0	*D* ^a^ ≥ 1			
*d* ^b^ = 0	119 (64.7%)	59 (13.8%)	<0.01	11.4	7.61–17.22
*d* ^b^ ≥ 1	65 (35.3%)	369 (86.2%)			

^a^ *D* = decayed PFMs, ^b^*d* = decayed primary second molar, ^c^*χ*^2^ test, ^d^OR unadjusted odds ratio.

**Table 5:** The neighboring teeth correlation in evaluated molars (Spearman's correlation coefficient).

Molars	*r*_s_^a^	*p* ^*∗*^
16/55	0.01	0.994
26/65	−0.16	0.696
36/75	−0.03	0.360
46/85	−0.07	0.050

^a^ *r*_s_ = Spearman's rho.  ^*∗*^*p* > 0.05 nonsignificant.

## Data Availability

All data generated or analyzed during this study are included in this article. Further inquiries can be directed to the corresponding author.
